# Patient-physician agreement on tobacco and alcohol consumption: a multilevel analysis of GPs’ characteristics

**DOI:** 10.1186/s12913-015-0767-6

**Published:** 2015-03-18

**Authors:** Jean-Laurent Thebault, Hector Falcoff, Madeleine Favre, Frédérique Noël, Laurent Rigal

**Affiliations:** Department of General Practice, Sorbonne Paris Cité, Paris Descartes University, Paris, France; Inserm, Centre for Research in Epidemiology and Population Health, U1018, Gender, Health, Sexuality Team, Villejuif, France; Paris Sud University, UMRS 1018, Villejuif, France; Ined, Paris, France; Société de Formation Thérapeutique du Généraliste, Paris, France

**Keywords:** General practice, Alcohol drinking, Smoking, Agreement, Medical records

## Abstract

**Background:**

Data about tobacco and alcohol consumption are essential in many types of studies. These data can be obtained by directly questioning patients or by using the information collected from physicians. Agreement between these two sources varies according to the characteristics of patients but probably also those of physicians. The purpose of this study was to analyze the characteristics of general practitioners (GPs) associated with agreement between them and their patients about the patients’ consumption of alcohol and tobacco.

**Methods:**

Data came from an observational survey among GPs who were internship supervisors in the Paris metropolitan area. Fifty-two volunteer GPs completed a self-administered questionnaire about the organization of their practice and their training. For each GP, a random sample of 70 patients, aged 40 to 74 years, answered questions about their personal tobacco and alcohol consumption. GPs simultaneously answered similar questions about each patient. We used a mixed logistic model to assess the association between physicians’ characteristics and agreement for patients’ smoking status and alcohol consumption.

**Results:**

Data were collected from both patient and physician for 2599 patients. The agreement between patients and their physicians was 60.4% for smoking status and 48.7% for alcohol consumption. Physicians with continuing medical education in management of smokers and those reporting specific skill in managing hypertension had the best agreement for smoking. Physicians who taught courses at the university medical school and those reporting specific skill in managing alcoholism had the best agreement for alcohol consumption.

**Conclusions:**

Agreement increases with physicians’ training and skills in management of patients with tobacco and alcohol problems. It supports the importance of professional training for improving the quality of epidemiologic data in general practice. Researchers who use GPs as a source of information about patients’ tobacco and alcohol consumption must assess the physicians’ characteristics.

## Background

Tobacco and alcohol consumption are two major causes of morbidity and mortality. Worldwide, the mortality attributable to smoking has been estimated at 12% [1] and that attributable to alcohol at 3.8% [2]. Data about consumption of these products, which are risk factors for numerous diseases, are essential in many types of studies, especially epidemiologic, as a variable of interest or for adjustment [3]. These data can be obtained by directly questioning patients or by collecting the information from physicians [4,5]. General practitioners (GPs) are often questioned about this [6,7]. The information collected from physicians and patients is not always identical. They agree more closely for smoking than for alcohol consumption [7,8]. In addition, agreement for smoking varies according to patients’ characteristics: it is poorer for patients who are younger, male, or more socially disadvantaged [3].

Few studies [9,10] have examined the variations in agreement according to physicians’ characteristics. Nonetheless, when data are collected from physicians (for reasons of simplicity or if the patients cannot be questioned directly), knowing these characteristics would make it possible to identify the GPs with the most reliable information about consumption (that is, the closest to the patient-reported data). Some characteristics of practice organization, such as the number and types of professionals in the office, have already been studied, but no significant associations have been identified [9]. Moreover, agreement differed according to type of practice organization (medical group *vs* independent practice association) in a US study, although this study did not consider tobacco and alcohol consumption [10]. To the best of our knowledge, other important organizational aspects, including the duration of consultations, have not been analyzed. Moreover, we might suppose that physicians’ training, especially in managing smoking and alcoholism, might affect the agreement rate, but this hypothesis has never been tested.

The objective of this study was to analyze whether and if so what GP characteristics (related to their organization of work and their initial and continuing training) are associated with agreement between patients and physicians about the patients’ use of tobacco and alcohol.

## Methods

### Study design

This study is an ancillary analysis of data from an observational survey named *Prev Quanti* [11]. This survey was initially designed to document social inequalities in preventive care (screening for breast, cervical, and colorectal cancer, tobacco and alcohol consumption, and cardiovascular risk) provided by GPs. To have a sample size large enough to be able to study the cancer screening tests recommended for patients aged 50–74 years, while still being able to analyze young patients at low cardiovascular risk, we chose to include only patients aged 40 to 74 years. A power calculation determined that we would require 50 GPs and 70 patients per GP to be able to demonstrate social gradients for the types of preventive care studied. The *Prev Quanti* study was conducted in 2008–09 among GPs who supervised students training in general practice during internships at their offices. We used email and telephone to recruit from among the 215 GPs working with two medical school departments of general practice in the Paris metropolitan area (who were paid 300 € for work estimated to take around 10 hours). For each participating GP, a random sample of 35 men and 35 women aged 40 to 74 years was drawn from their patient list (patients who had reported them to be their regular GP), furnished by the national health insurance fund. There were no exclusion criteria.

### GPs’ characteristics

The GPs’ characteristics were collected by a self-administered questionnaire. The first group of characteristics concerned the organization of their practices: fixed fees or authorization for fees beyond the amount reimbursed by the national health insurance fund, group or solo practice, mean number of consultations per week, mean duration of consultations, percentage of consultations by appointment (compared with open hours), use of computerized medical files, and use of automatic reminders. The second group of characteristics concerned their initial and continuing training: time since completion of medical school, teaching medical school courses (above and beyond supervising internships), participation in peer groups, responsibility in an organization offering continuing medical education (CME), participation in a CME course or a study on any or more of six prevention themes (hypertension, smoking, alcoholism, and screening for breast cancer, colon cancer, and cervical cancer), and reporting specific skill for each of these themes.

### Patients’ characteristics

All of the included patients received a questionnaire from their GP, which asked about their smoking status, alcohol consumption, and social situation. They were asked to complete and return it to the GP. At the same time, the GPs were asked to complete a form covering the same data for each patient, using the information in their medical files or what they knew about the patient, even if not mentioned in the medical file.

Patients’ smoking status, collected from the patient and the physician, was classified in three categories: current smoker, ex-smoker and non-smoker. Neither group’s report of smoking status was considered a gold standard.

The binary variable of agreement about smoking status was defined as positive when both the patient and the physician placed the patient in the same category. The physician, unlike the patient, could answer “do not know” about the patient’s smoking status. In this case, the response was considered discordant regardless of the patient’s response.

The variable of agreement about alcohol consumption was defined similarly to that for smoking but depended on the patient’s level of alcohol consumption, classified in four categories: abstinent, moderate consumption (≤210 g weekly for men and ≤ 140 g for women), occasional excessive consumption (≥50 g on a single occasion) and regular excessive consumption (>210 g weekly for men and > 140 g for women). This classification was determined by the alcohol consumption reported, and the cut-off values are those set by the French Society of Alcohol Studies [12].

### Statistical analysis

We describe below only the analyses for smoking but the same analytic strategy was used for alcohol consumption. We first calculated the agreement among all patients for whom this calculation was possible (that is, in including all the physicians with “do not know” responses). Next we calculated the agreement and a weighted kappa coefficient (quadratic weighting) [13], only among patients whose physicians did not answer “do not know”, as is usual in the literature [7,8].

We used a mixed logistic model [14] with a random intercept [15,16] to assess the association between physicians’ characteristics and agreement for smoking status (the dependent variable). The structure of our data (that is, that patients are grouped by physicians) mandates the use of these statistical models [17] to obtain unbiased estimators [18]. These models simultaneously include fixed indicators that enable ORs to be calculated, as in the standard models, and a random indicator, inter-GP variance, which allows us to calculate variations in the agreement rates between GPs [19].

We performed a univariate and then a multivariate analysis, adjusted for patient’s sex, age (40–50 years, 51–60, 61 to >70), duration of time seeing this GP (<1 year, 1–5, >5), number of consultations with the GP during the past year (<3, ≥3) and occupation, as well as for the physician’s sex [20] and age (in quartiles). We included in a multivariate model all of the GPs’ characteristics significantly associated with agreement for smoking status in the univariate analysis, at a threshold of 20%. We then removed the GP characteristics according to a backward stepwise strategy, with a threshold of 5%, to obtain the final model.

At the end, to evaluate the percentage of inter-GP variance (s^2^) explained by the GP characteristics in the final model, we constructed another multivariate model (called the reduced final model) containing all patient characteristics and the GPs’ age and sex but without any of the other GP characteristics present in the final model. The percentage of reduction of the inter-GP variance between these two models was calculated as follows: Δs^2^ = 100.(s^2^_reduced final model_ - s^2^_final model_)/s^2^_reduced final model_.

This calculation allowed us to quantify the effect of GPs’ characteristics on the variations in the agreement rate between GPs, independently of any effect due to the composition of their practice (patient load) [17].

All statistical analyses were performed with R software, version 2.14.1, and SAS software, version 9.3. The study was approved by the advisory committee on information treatment for health research (*Commission nationale de l’informatique et des libertés*), and all patients signed informed consents.

## Results

The first 52 GPs who volunteered to participate were included in the study. Of the 3640 randomly selected patients, the return rate for physician questionnaires was 98.9% (n = 3600), while the patient participation rate was 71.6% (n = 2605). Finally, data were collected from both patient and physician for 71.4% (n = 2599) of the patients included.

The physicians’ mean age was 55 years (SD = 6), and 63% of them were men (Table 1). The mean duration of consultations was 21 minutes (SD = 4.8), and physicians saw 92 (SD = 23) patients weekly, on average.

**Table 1:** **Patient (n = 2599) and GP (n = 52) characteristics**

**Patient characteristics**	**n (%)**
Age (years) 40-50	1027 (39)
51-60	829 (32)
61-70	598 (23)
>70	145 (6)
Male	1255 (48)
Duration of time seeing the GP (years) [0, 1[	168 (6)
[1-5[	846 (33)
>5	1558 (60)
Number of consultations during the past year ≤ 3	1451 (55)
>3	1061 (40)
Occupational group Shopkeepers and crafts workers	114 (4)
Professionals and managers	1133 (44)
Intermediate white-collar workers	502 (19)
Office, sales, and service employees	446 (18)
Skilled or unskilled manual workers	214 (8)
**GP characteristics**	n (%)
Age (years) ≤50	570 (22)
51-56	638 (25)
57-61	827 (32)
≥62	564 (22)
Male	33 (63)
Participation in a CME^*^ course about managing: Hypertension	33 (63)
Smoking cessation	23 (44)
Alcoholism	23 (44)
Reporting specific skills about managing: Hypertension	15 (29)
Smoking cessation	12 (23)
Alcoholism	12 (23)
Teaching medical school course	32 (61)
Responsibility in a organisation providing CME	14 (27)
Participation in peer groups	33 (63)
Group practice	37 (71)
Fixed fees	33 (63)
≥80 % consultations by appointment	41 (79)
Using computerised medical files	46 (88)
Using automatic reminders	15 (28)

^*^CME: continuing medical education.

The patients’ mean age was 54 years (+/−9) (Table 1). The median duration of the physician-patient relationship was 7 years (interquartile range (IQR) = 3-11) and the median number of consultations during the past year 3 (IQR = 1-5).

The patients’ non-response rate to the smoking questions was 0.3% (n = 8), and to the drinking questions, 23% (n = 596).

Overall, 27.6% of the patients reported they smoke (Table 2), and 23.9% reported occasional or regular excessive alcohol consumption (Table 3).

**Table 2:** **Patients’ smoking status according to patients and physicians**

**Smoking status**		**According to patients**			
**n**					
***%***		**Non-smoker**	**Ex-smoker**	**Current smoker**	**Total**
**According to physicians**	Non-smoker	707	177	73	957
		*27.3*	*6.8*	*2.8*	*36.9*
	Ex-smoker	81	407	81	569
		*3.1*	*15.7*	*3.1*	*22,0*
	Current smoker	34	59	450	543
		*1.3*	*2.3*	*17.4*	*21,0*
	Do not know	284	127	111	522
		*11,0*	*4.9*	*4.3*	*20,1*
	Total	1106	770	715	2591
		*42.7*	*29.7*	*27.6*	*100*

**Table 3:** **Patients’ alcohol consumption according to patients and physicians**

**Alcohol consumption**		**According to patients**				
**n**						
***%***		**Abstinent**	**Moderate consumption**	**Occasional excessive consumption**	**Regular excessive consumption**	**Total**
**According to physicians**	Abstinent	101	138	10	21	270
		*5,0*	*6,9*	*0.5*	*1,0*	*13.5*
	Moderate consumption	112	795	123	111	1141
		*5,6*	*39.7*	*6.1*	*5.5*	*57,0*
	Occasional excessive consumption	3	61	20	37	121
		*0.1*	*3,0*	*1,0*	*1.8*	*6.0*
	Regular excessive consumption	13	41	18	60	132
		*0.6*	*2,0*	*0.9*	*3,0*	*6.6*
	Do not know	50	211	39	39	339
		*2,5*	*10,5*	*1.9*	*1.9*	*16,9*
	Total	279	1246	210	268	2003
		*13.9*	*62.2*	*10.5*	*13.4*	*100*

For smoking, the mean agreement rate was 60.3%. This rate varied significantly between physicians, ranging from 41.2% to 77.8% for the 10^th^ and 90^th^ percentiles of the distribution (s^2^ = 0.63). Among patients whose physician did not answer “do not know”, agreement was 75.6% and the weighted kappa coefficient was 0.70. Agreement for smoking status was higher among physicians who had done a CME course about managing smoking cessation and those who reported specific skill in managing hypertension (Table 4). The reduction in the inter-GP variance because of these two GP characteristics was 21%.

**Table 4:** **Association between GPs’ characteristics& and agreement for smoking status (n = 2322)**

	**Univariate analysis**			**Multivariable analysis** ^*^		
	**OR**	**95% CI**	***P***	**OR**	**95% CI**	***P***
Age (years, ref :≤ 50)			0.90			0.94
51-56	1.00	0.57-1.74		0.97	0.58-1.63	
57-61	1.01	0.61-1.69		1.02	0.63-1.66	
≥62	0.84	0.48-1.48		0.87	0.51-1.51	
Male	1.28	0.87-1.89	0.22	0.71	0.48-1.05	0.088
Teaching medical school course	1.43	0.98-2.08	0.063			
Responsibility in an organization providing CME^#^	1.66	1.10-2.50	0.015			
Participation in a CME^#^ about managing smoking cessation	1.54	1.07-2.21	0.021	1.71	1.18-2.49	0.005
Participation in a CME^#^ about colon cancer screening	1.38	0.92-2.06	0.12			
Participation in a study about colon cancer screening	0.73	0.46-1.17	0.19			
Participation in peer groups	1.42	0.97-2.08	0.072			
Reporting specific skills in managing hypertension	1.32	0.87-1.99	0.19	1.72	1.11-2.65	0.015
Reporting specific skills in managing smoking cessation	1.35	0.86-2.10	0.19			
Reporting specific skills about cervical cancer screening	1.35	0.90-2.04	0.15			
Using computerized medical files	0.68	0.38-1.22	0.19			

Inter-GP variance: s^2^ = 0.31, Hosmer-Lemeshow test of goodness of fit: *P* = 0.11.

& Only GPs characteristics with *P* ≤ 0.20 in univariate analysis are shown.

^*^adjusted for patient’s sex, patient’s age, duration of time seeing this GP, number of consultations during the past year, patient’s occupation, GP’s age and GP’s sex.

^#^CME: Continuing medical education.

For drinking, the mean agreement rate was 48.7%. This rate again varied significantly between physicians, from 36.0% to 60.8% for the 10^th^ and 90^th^ percentiles of the distribution (s^2^ = 0.40). Among patients whose physician did not answer “do not know”, the agreement was 58.6% and the weighted kappa coefficient was 0.33. Agreement for alcohol consumption was higher among GPs teaching courses at the medical school and those GPs reporting specific skill in managing alcoholism (Table 5). The reduction in the inter-GP variance because of these two GP characteristics was 29%.

**Table 5:** **Association between GPs’ characteristics& and agreement for alcohol consumption (n = 1827)**

	**Univariate analysis**			**Multivariable analysis** ^*^		
	**OR**	**95% CI**	***P***	**OR**	**95% CI**	***P***
Age (years, ref:≤ 50)			0.80			0.32
51-56	0.89	0.59-1.34		0.74	0.49-1.13	
57-61	1.03	0.70-1.51		0,78	0.52-1.12	
≥62	1.09	0.72-1.66		1.03	0.69-1.55	
Male	0.83	0.62-1.10	0.20	0.85	0.64-1.12	0.25
Teaching medical school course	1.35	1.02-1.78	0.036	1.42	1.06-1.90	0.019
Participation in a study about cervical cancer screening	1.41	0.95-2.10	0.091			
Participation in peer groups	1.27	0.96-1.69	0.099			
Reporting specific skills in managing smoking cessation	1.42	1.04-1.95	0.030			
Reporting specific skills in managing alcoholism	1.30	0.94-1.80	0.12	1.57	1.09-2.27	0.015
Reporting specific skills in cervical cancer screening	1.27	0.94-1.71	0.12			
Reporting specific skills in breast cancer screening	1.25	0.92-1.70	0.16			
≥80% consultations by appointment	1.46	1.04-2.03	0.027			

Inter-GP variance: s^2^ = 0.11, Hosmer-Lemeshow test of goodness of fit: *P* = 0.41.

& Only GPs characteristics with p ≤ 0.20 in univariate analysis are shown.

^*^adjusted for patient’s sex, patient’s age, duration of time seeing this GP, number of consultations during the past year, patient’s occupation, GP’s age and GP’s sex.

## Discussion

In our study, the agreement between patients and their physicians was about 60% for smoking status and almost 50% for alcohol consumption. Both varied significantly between the GPs. Physicians with a CME course in management of smoking cessation and those reporting specific skill in managing hypertension had the best agreement for smoking. Physicians who taught courses at the university medical school and those reporting specific skill in managing alcoholism had the best agreement for alcohol consumption. No characteristic of the organization of their practice was significantly associated with agreement.

To our knowledge, this study is the first to have tested the association between a large group of physicians’ characteristics related to their practice organization and medical education and agreement about smoking and alcohol consumption.

Another strength of our study is the good level of patient participation. This is explained in part by specific characteristics of the physicians who recruited them. The fact that the request for participation came from their regular GP [21] and that this physician had an association with the university [22] probably gave them confidence.

To assess the physicians’ knowledge of their patients’ smoking status and alcohol use, we used what the GPs actually knew about their patients, even if it was not written in the medical files. That is, GPs in France mainly use their files as practice reminders rather than exhaustive data collections or protection against malpractice suits: they frequently do not include everything they know about the patient (especially the things they know well and are unlikely to forget) [23]. We also calculated agreements based only on what was recorded in the medical files. These agreement rates were lower (39.4% for smoking status and 13.3% for alcohol use, results not shown) than those we obtained in the principal analyses.

In addition to the portion of disagreement resulting from our treating physicians’ “do not know” responses as disagreement, several other points may explain some of this discordance between patients and physicians. Patients generally underestimate their consumptions during questionnaire surveys [24]. But because the patients had to send the questionnaire back to the GP, they probably responded as they would have during a consultation with him or her. From their perspective, some physicians might also have assumed under-reporting by their patients and accordingly estimated consumption at a level higher than reported.

Moreover, some patients might have modified their consumptions between their last consultation and the time that they completed the questionnaire, in particular those whom the doctors had not seen recently. To try to take time since the last consultation into account (and thus limit the measurement bias for the agreement), we adjusted our analyses for the number of consultations during the previous year (which was significantly associated with agreement, results of adjustment not shown). We also conducted a sensitivity analysis, excluding patients who had not been seen in the past year (16.7% of patients); its results were nearly identical to those presented. Agreement was 62.7% for smoking status and 50.0% for alcohol consumption. The results of the final models also remained very similar in terms of the significance of the variables, and the direction and intensity of the associations.

Another particularity of our study is that we collected the doctor’s opinion (as well as the file information). This opinion might be affected by memory errors or based on prejudices [25].

Finally, some part of the disconcordance may be due to the fact that physicians do not regularly collect updated information on these topics, although such updating is recommended [23].

For some GPs, agreement might have been better because they had patients with the characteristics associated with better agreement and not because of their own characteristics. Agreement is best, for example, for the most advantaged patients [3]. Physicians whose patients were largely well-off could thus have had better agreement that was not associated with their own characteristics. We took this confounding into account by using hierarchical models adjusted for different patient characteristics (such as occupation) that are known to be associated with agreement.

Two hypotheses might explain why we found so few GP characteristics associated with agreement. Our sample of physicians might be too homogeneous. All the participating GPs also supervise internships, which tends to encourage them to follow the clinical practice guidelines [23] that mandate the inclusion of smoking and alcohol status in medical files. Nonetheless this theory seems to be contradicted by the great variability observed between the GPs. More probably, the relatively low number of physicians included in the study limited the power of our statistical tests: in hierarchical models with random physician effects, power is linked above all else to the number of physicians analyzed [24,26].

The generalizability of our results might also be questioned, especially because all the participating GPs supervise interns. However, the characteristics of the participating GPs are not very different from those of all French GPs, particularly with regard to age, sex, fixed fees or not, and solo or group practice [27], and the agreement we observed for smoking status and alcohol consumption was quite similar to the agreement reported in the literature (as discussed below). Moreover, the association measures between agreement and GPs’ characteristics reported here do not appear likely to be influenced by internship supervision.

The kappa for smoking status that we observed (0.70) corresponds to substantial agreement [28].

This result is consistent with previous studies that have found kappa coefficients between 0.46 and 0.83 [7,8,29,30]. The kappa for alcohol consumption (0.33) corresponds to fair agreement and is lower than that reported in the study by Mant et al. (0.53) [7]. This difference is probably due to the existence of the category of occasional excessive consumption, which the physicians selected rather rarely: only 9.5% of the patients who reported such consumption were so classified by their GPs (Table 3).

We found few reports to compare with our results about the physicians’ characteristics associated with agreement. The study by Ferrante et al., which is the closest to ours, did not look at alcohol consumption, nor did it find the organizational characteristics of the practice to be associated with agreement about smoking [9].

Physicians trained in managing patients who smoke had the best agreement rate, probably because they are more involved in screening and helping their patients to stop smoking [31]. Similarly, physicians skilled in managing patients with drinking problems feel that they can appropriately be interested in their patients’ consumption. It is well known that one of the major obstacles to screening for alcoholism is physicians’ feeling of impotence in this field and their lack of training [32].

## Conclusions

Agreement increases with physicians’ training and skills in management of patients with tobacco and alcohol problems. Our results thus underline the importance of professional training, which still requires improvement. Our results should also induce researchers to look at physicians’ characteristics when they ask GPs about their patients’ smoking status and alcohol consumption. The data in studies of phenomena associated with use of these substances would probably be of higher quality if the researchers recruited as investigators physicians trained or specifically skilled in these domain, for example, through a CME organization or a relevant care network.

## Abbreviations

CCTIRS: Advisory committee on data processing in health research (Comité consultatif sur le traitement de l’information en matière de recherche dans le domaine de la santé)
CME: Continuing medical education
GP: General practitioner
IQR: Interquartile range
SD: Standard deviation
SFTG: French society of generalist treatment (Société de formation thérapeutique du généraliste)
WHO: World Health Organization

## References

[1] World Health Organization (2012). WHO global report on mortality attributable to tobacco.

[2] World Health Organization, Management of Substance Abuse Team (2011). Global Status Report on Alcohol and Health.

[3] Newell SA, Girgis A, Sanson-Fisher RW, Savolainen NJ (1999). The accuracy of self-reported health behaviors and risk factors relating to cancer and cardiovascular disease in the general population: a critical review. Am J Prev Med.

[4] Pakhomov SV, Jacobsen SJ, Chute CG, Roger VL (2008). Agreement between patient-reported symptoms and their documentation in the medical record. Am J Manag Care.

[5] Murray RL, Coleman T, Antoniak M, Fergus A, Britton J, Lewis SA (2008). The potential to improve ascertainment and intervention to reduce smoking in Primary Care: a cross sectional survey. BMC Health Serv Res.

[6] Simpson CR, Hippisley-Cox J, Sheikh A (2010). Trends in the epidemiology of smoking recorded in UK general practice. Br J Gen Pract J R Coll Gen Pract.

[7] Mant J, Murphy M, Rose P, Vessey M (2000). The accuracy of general practitioner records of smoking and alcohol use: comparison with patient questionnaires. J Public Health Med.

[8] Wilson A, Manku-Scott T, Shepherd D, Jones B (2000). A comparison of individual and population smoking data from a postal survey and general practice records. Br J Gen Pract.

[9] Ferrante JM, Ohman-Strickland P, Hahn KA, Hudson SV, Shaw EK, Crosson JC (2008). Self-report versus Medical Records for Assessing Cancer-Preventive Services Delivery. Cancer Epidemiol Biomarkers Prev.

[10] Tisnado DM, Adams JL, Liu H, Damberg CL, Hu A, Chen W-P (2007). Does concordance between data sources vary by medical organization type. Am J Manag Care.

[11] Société de formation thérapeutique du Généraliste (2010). Mobiliser les médecins traitants franciliens pour réduire les inégalités de prévention et dépistage.

[12] Société française d’alcoologie (2003). Alcohol misuse except alcohol addiction: use and misuse. Alcoologie Addictologie.

[13] Cohen J (1960). A Coefficient of Agreement for Nominal Scales. Educ Psychol Meas.

[14] Coughlin SS, Pickle LW, Goodman MT, Wilkens LR (1992). The logistic modeling of interobserver agreement. J Clin Epidemiol.

[15] Snijders TAB, Bosker R (2011). Multilevel Analysis: An Introduction to Basic and Advanced Multilevel Modeling.

[16] Raudenbush SW, Bryk AS (2001). Hierarchical Linear Models: Applications and Data Analysis Methods.

[17] Diez R (2002). A glossary for multilevel analysis. J Epidemiol Community Health.

[18] Moerbeek M, van Breukelen GJP, Berger MPF (2003). A comparison between traditional methods and multilevel regression for the analysis of multicenter intervention studies. J Clin Epidemiol.

[19] Merlo J, Chaix B, Yang M, Lynch J, Råstam L (2005). A brief conceptual tutorial of multilevel analysis in social epidemiology: linking the statistical concept of clustering to the idea of contextual phenomenon. J Epidemiol Community Health.

[20] Soto CM, Kleinman KP, Simon SR (2002). Quality and correlates of medical record documentation in the ambulatory care setting. BMC Health Serv Res.

[21] Rigal L, Saurel-Cubizolles M-J, Falcoff H, Bouyer J, Ringa V (2011). Do social inequalities in cervical cancer screening persist among patients who use primary care? The Paris Prevention in General Practice survey. Prev Med.

[22] Kaguelidou F, Amiel P, Blachier A, Iliescu C, Rozé J-C, Tsimaratos M (2013). Recruitment in pediatric clinical research was influenced by study characteristics and pediatricians’ perceptions: a multicenter survey. J Clin Epidemiol.

[23] Agence nationale d’accréditation et d’évaluation en santé (ANAES): La Tenue Du Dossier Médical En Médecine Générale: État Des Lieux et Recommandations. Paris, France; 1996

[24] Snijders TAB, Bosker RJ (1993). Standard errors and sample sizes for two-level research. J Educ Behav Stat.

[25] Van Ryn M, Burke J (2000). The effect of patient race and socio-economic status on physicians’ perceptions of patients. Soc Sci Med 1982.

[26] Kreft IGG, de Leeuw J (1998). Introducing Multilevel Modeling.

[27] Institut national de prévention et d’éducation pour la santé (2011). Baromètre santé médecins généralistes 2009.

[28] Landis JR, Koch GG (1977). The measurement of observer agreement for categorical data. Biometrics.

[29] Eze-Nliam C, Cain K, Bond K, Forlenza K, Jankowski R, Magyar-Russell G (2012). Discrepancies between the medical record and the reports of patients with acute coronary syndrome regarding important aspects of the medical history. BMC Health Serv Res.

[30] Tisnado DM, Adams JL, Liu H, Damberg CL, Chen W-P, Hu FA (2006). What is the concordance between the medical record and patient self-report as data sources for ambulatory care?. Med Care.

[31] Stead M, Angus K, Holme I, Cohen D, Tait G (2009). Factors influencing European GPs’ engagement in smoking cessation: a multi-country literature review. Br J Gen Pract.

[32] Blanquet M, Peyrol MF, Gerbaud L, Morel F, Maradeix B, Llorca P-M (2014). Tackling the alcohol issue in France. Br J Gen Pract.

